# Cerebral and intestinal Doppler patterns according to patent ductus arteriosus shunt characteristics in preterm infants

**DOI:** 10.1038/s41372-025-02505-9

**Published:** 2025-11-24

**Authors:** Joshua Mifflin, Marjorie Makoni, Trassanee Chatmethakul, Patrick J. McNamara, Adrianne R. Bischoff

**Affiliations:** 1 University of Iowa, Department of Pediatrics, Iowa City, IA USA; 2 University of Oklahoma Health, College of Medicine, Department of Pediatrics, Section of Neonatal-Perinatal Medicine, Oklahoma City, OK USA; 3 University of Iowa, Department of Pediatrics, Division of Neonatology, Iowa City, IA USA; 4 University of Iowa, Department of Internal Medicine, Iowa City, IA USA

**Keywords:** Paediatrics, Cardiovascular diseases

## Abstract

**Background:**

A hemodynamically significant patent ductus arteriosus (hsPDA) is common in preterm infants and may impact systemic and cerebral blood flow. This study explores Doppler flow patterns in systemic arteries including celiac artery/trunk (CeT), superior mesenteric artery (SMA) and middle cerebral artery (MCA) in preterm neonates with varying PDA shunt patterns, utilizing targeted neonatal echocardiography (TNE).

**Methods:**

Retrospective multicenter study of preterm infants (born <32 weeks gestation, <40 days of life) who underwent comprehensive TNE. Infants were categorized into 3 groups based on PDA shunt characteristics as follows: (1) No transductal shunt or trivial PDA with PDA score <6, (2) Left-to-right PDA (L-R PDA) and PDA score ≥8 and (3) Bidirectional PDA shunt with at least 10% of time spent right-to-left (R-L) and PDA score <6.

**Results:**

In total, 86 patients with a median [IQR] gestational age of 26.1 weeks [23.8–27.7] weeks and birth weight of 767 [596, 1016] grams were included and classified into 3 groups: No PDA (*n* = 29), hsPDA (*n* = 48) and bidirectional PDA (*n* = 27). Abnormal systemic arterial Doppler patterns (absent or reversed diastolic flow in the Celiac artery) were present in 2 (5.7%), 15 (44.1%), and 1 (5.9%) of the patients in the no PDA, hsPDA, and bidirectional PDA groups respectively. Diastolic flow reversal in any of the systemic vessels evaluated was demonstrated only in the hsPDA group.

**Conclusion:**

This physiologic study demonstrates altered systemic Doppler patterns, which suggests a decreased ability to preserve lower body blood flow in premature infants with hsPDA. Further prospective studies are warranted to describe the association of these physiologic findings with clinical outcomes such as necrotizing enterocolitis.

**Clinical Trial Registration (if any):**

None (not applicable).

## Introduction

A hemodynamically significant patent ductus arteriosus (hsPDA) in preterm infants is considered a major contributor to abnormal systemic and cerebral blood flow [1–5]. Though the clinical significance of left to right hsPDA shunt is yet to be clearly delineated [6, 7], compromised cerebral and systemic blood flow have been posited as contributing factors to PDA-associated morbidities, including necrotizing enterocolitis (NEC), chronic lung disease (CLD), and intraventricular hemorrhage [8–10]. Traditional clinical markers, such as blood pressure or murmur, are confounded by their poor specificity and interobserver agreement [11]. Systemic circulation in hsPDA physiology is theorized to be compromised by a “diastolic steal” or a “diastolic runoff” phenomenon [12, 13]. This, in turn, may compromise systemic vessel flow volumes, depending on the magnitude and size of the shunt [11, 14].

The use of targeted neonatal echocardiography (TNE) performed by trained neonatologists has revolutionized the care of premature babies by providing physiology-based and individualized care [14, 15]. Although the ability to determine shunt significance by echo has improved, clearly defining the relationship between the magnitude of shunt volume and systemic steal remains a knowledge gap. TNE is a potential tool for evaluating flow dynamics and the potential flow-driven morbidities affected by the severity of the ductal shunt [14].

Previous studies have consistently demonstrated that infants with a hsPDA and retrograde diastolic flow in the descending aorta have higher left ventricular output [16, 17]. Absent or reversal of flow during diastole can occur in the post-ductal arteries (descending aorta [Dao], celiac artery/trunk [CeT], superior mesenteric artery [SMA]), as well as the pre-ductal circulation, including the middle cerebral artery (MCA) [1–5, 18, 19].

In this physiologic study, our primary aim was to characterize the Doppler flow patterns of the MCA, DAo, CeT, and SMA according to PDA shunt patterns. We hypothesized that infants with hsPDA will have higher incidence of retrograde diastolic blood flow in the DAo, SMA, CeT and MCA compared to those with bidirectional shunt and no transductal shunt.

## Methods

We conducted a retrospective multicenter cohort study of preterm infants born <32 weeks gestation who underwent a comprehensive TNE in the first six postnatal weeks. Neonates admitted to the neonatal intensive care unit at the University of Iowa Stead Family Children’s Hospital and University of Oklahoma Children’s Hospital, between January 1st, 2019, and April 30th, 2022, were included. The study was approved by each Institutional Research Ethics Board with waiver of consent. Exclusion criteria included infants identified with congenital heart disease including atrial and/or ventricular septal defects >3 mm, congenital diaphragmatic hernia, and infants that did not fit into any of the predefined PDA group classifications.

### Data collection

Clinical data was collected from electronic medical charts. The following data were collected: (i) neonatal demographics including gender, gestational age, birthweight, size for gestational age, postmenstrual age, and weight at the time of TNE, (ii) delivery details including mode of delivery, APGAR scores, use of surfactant and (iii) cardiorespiratory status at time of assessment including ventilation mode and parameters, blood gas results within 6 hours of TNE assessment, vasopressors/inotropes and pulmonary vasodilator use at the time of the assessment.

#### Echocardiography assessment

Comprehensive TNE to screen for hsPDA in infants born less than or equal to 32 weeks were performed and interpreted by neonatologists with hemodynamic expertise and comprehensive training in image acquisition and interpretation at both centers [20]. All TNE evaluations were performed according to a standardized protocol which includes approximately 80–100 images and takes up to 15–20 min. All echocardiography analyses were performed, using a dedicated workstation (EchoPAC version BT10; GE Medical Systems, Milwaukee, USA), by two trained investigators (JM and MM). A standardized protocol sequence was used for all patients. If the patient had multiple TNEs, only one exam was used in the study. All measurements were averaged over three consecutive cardiac cycles. Flow across other shunts (i.e. atrial communication) was identified using color Doppler imaging.

##### Left heart volume evaluation

In the apical four-chamber view, analyses of the pulmonary D wave velocity, mitral peak E wave, and isovolumic relaxation time (IVRT) were completed with measurements according to published standards [4, 21–24]. The LA:Ao was assessed with M-mode in the parasternal long axis with the cursor placed at the plane of the aortic valve hinges maximizing Left atrium (LA) diameter within a plane perpendicular to the aortic wall at the level of the valve.

##### Left and right ventricular output evaluation

Left ventricular output (LVO) was obtained by placing a pulsed-wave Doppler (PWD) sample gate perpendicular to the aortic valve in the apical five-chamber view, with the angle of insonation parallel to the left ventricular outflow tract. No angle correction software was performed and only images with less than 5 degrees deviation from the flow direction were used. Three consecutive cardiac cycles were measured and averaged. The area under the waveform of the aortic systolic beat was traced to obtain the Velocity Time Integral (VTI) and the heart rate. The annulus of the aortic valve was measured, from the parasternal long axis, between hinge points with the valve open at the end of ejection. LVO (expressed in mL/min/kg) was calculated by multiplying the aortic cross-sectional area (CSA) [calculated as: (aortic radius^2^ x π)] multiplied by VTI and heart rate and indexed to weight (kg) [21, 24].

Right ventricular output (RVO) was measured using a similar approach. The right ventricular VTI was obtained by placing the PWD sample volume at the level of the pulmonary artery valve either in the parasternal short or long axis, whichever view was more parallel to the direction of blood flow (angle of insonation with less than 5 degrees deviation from the direction of flow). Pulmonary artery diameter was measured in the same view, between hinge points, at the end of flow ejection. In the presence of diastolic turbulence due to left-to-right PDA shunt, an attempt was made to trace the main envelope of the RVO, excluding the diastolic flow disturbance.

##### PDA evaluation and group classification

Comprehensive PDA shunt volume evaluation was done utilizing the American Society of Echocardiography measurement guidelines [14]. The PDA internal diameter was measured at the narrowest portion on the pulmonary end, using 2-D imaging. The pattern of flow was assessed with both color and PWD. Bidirectional ductal shunts were assessed according to the proportion of time of left-to-right and right-to-left flow based on Doppler tracing. PDA scores were adapted from Rios et al. [25], and included PDA size indexed to weight, mitral valve E-wave velocity, pulmonary vein D-wave velocity, IVRT, LVO, LA:Ao, and presence/absence of diastolic flow reversal in the DAo, CeT, and/or MCA [Supplementary Table 1], with maximum possible score of 14 points. A score below 6 indicates a low-volume, suggesting minimal hemodynamic impact and often favoring conservative management. Scores between 6 and 8 indicate moderate shunting, where medical therapy may be warranted, while scores above 8 denote high-volume shunts, typically necessitating intervention. PDA was considered hemodynamically significant if the diameter indexed to weight was at least 1.5 mm/kg and PDA score was at least 8. A PDA was considered insignificant if diameter was less than 1 mm/kg, less than 0.5 mm absolute size and PDA score was less than 6. These definitions were done a priori and did not include characteristics which could be deemed borderline between the different groups (i.e. PDA scores 6–7, etc). Patients were categorized into three groups as follows:

**Group 1:** No transductal shunt or trivial PDA (PDA diameter ≤1 mm/kg AND ≤ 0.5 mm absolute size AND PDA score <6)

**Group 2:** Left-to-right PDA (L-R PDA) shunts which were considered hemodynamically significant (PDA diameter >1.5 mm/kg AND PDA score ≥8)

**Group 3:** Bidirectional PDA shunt with at least 10% of time spent right-to-left (R-L) and PDA score <6 [22]. The inclusion of a low PDA score in this definition was to guarantee that the bidirectional shunt was driven by pulmonary vascular resistance rather than due to flow-driven increases in pulmonary artery pressure.

##### Systemic artery TNE evaluation

From the aortic arch view, the pre-ductal DAo antegrade VTI was obtained by placing a PWD sample volume in line with DAo, proximal to the PDA with care to minimize the angle of insonation to less than 5 degrees from the direction of flow. In a similar fashion, the PWD sample volume was then placed in the post-ductal DAo to obtain antegrade and retrograde (if present) VTIs [26, 27]. The approximate net post-ductal DAo VTI was calculated as follows: Post-ductal DAo antegrade VTI—Post-ductal DAo retrograde VTI. Absent diastolic flow in DAo is considered a normal variant and therefore not evaluated in this study.

The *celiac artery* Doppler profile was assessed from the subcostal sagittal view of the abdominal aorta aligned parallel with the PWD cursor to minimize the angle of insonation. Color Doppler was used to identify the celiac artery at its origin from DAo. PWD sample volume was placed within the celiac artery, near its origin to the aorta, parallel to the direction of flow. The *SMA* Doppler profile was assessed in a similar manner. The *MCA* Doppler profile was assessed from the left or right sphenoidal foramen using color Doppler. PW Doppler tracing was obtained with sample volume maintained within the MCA vessel in parallel alignment, minimizing the angle of insonation [28].

**Fig. 1:**
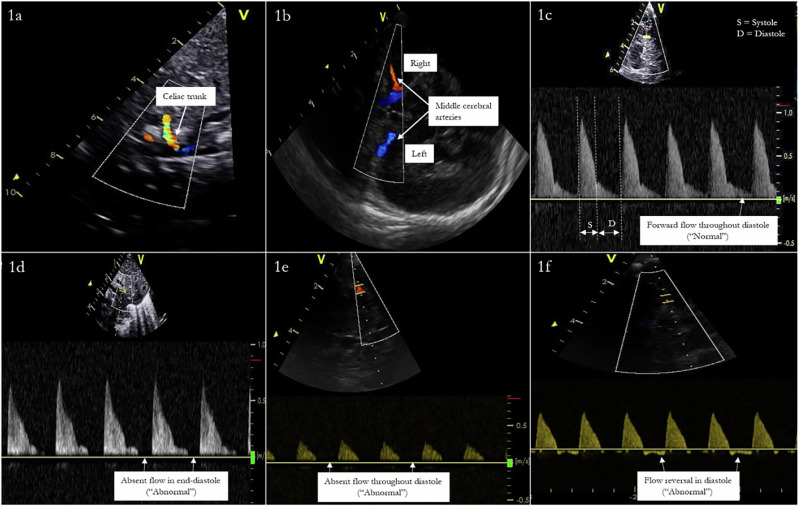
Doppler flow waveform of the celiac artery/trunk (CeT). Superior mesenteric artery (SMA), middle cerebral artery (MCA) and post-ductal descending aorta. Adapted from Keusters et al 2021 demonstrating the sagittal view of the celiac trunk (CeT) branching off the abdominal aorta (**a**), The Middle cerebral artery (MCA) (**b**). Forward flow in the MCA is depicted in (**c**) and abnormal flow (**d**) and absent end diastolic flow (**e**) and end diastolic flow reversal (**f**).

Antegrade VTI envelopes were interrogated by tracing the area under the waveform of the antegrade flow terminating at its return to the baseline, or onset of the next cardiac cycle; 3 sequential cardiac cycles were measured and averaged (Fig. 1) [26]. The maximum flow velocity (Vmax), minimum flow velocity (Vmin), and mean flow velocity (Vmean) were derived from their respective VTI envelopes and averaged from 3 consecutive beats. Absence or reversal of flow at any point during the cardiac cycle was reflected with a minimum velocity value of 0. The Pulsatility Index (PI) was calculated using these average values in the formula: (Vmax–Vmin) / Vmean. The Resistive Index (RI) was calculated using the average Vmax and Vmin values in the formula: (Vmax—Vmin) / Vmax [29]. Retrograde VTI envelopes were interrogated by tracing the area under the waveform of the retrograde flow terminating at its return to the baseline, or onset of the next cardiac cycle [26]. We attempted to obtain 3 measurements from successive beats if able—in some instances, true retrograde flow did not occur on every successive beat but instead with intermittent “absence of flow” characterization. In these instances, flow was qualitatively appraised as “retrograde” for this infant but would have those respective beat(s) quantitatively recorded as 0 to quantify the degree of retrograde flow more accurately. To account for diastolic flow reversal, we calculated a net post‑ductal descending aortic (DAo) VTI, defined as the antegrade VTI (time integral of forward velocities) minus the absolute value of the retrograde VTI (time integral of negative velocities during diastole). Measurements were averaged over 3–5 consecutive cardiac cycles. This derived parameter was used as a comparative index of effective forward momentum of flow and does not represent absolute volumetric blood flow.

### Outcomes

The primary outcome of abnormal systemic arterial Doppler pattern was defined as absent or reversed diastolic flow in the celiac artery. Secondary outcomes were absent or reversal of flow in the SMA and MCA, reversal of flow in the DAo, and changes in the measured VTI, Vmax, Vmin, Vmean and in the calculated PI and RI in the systemic Doppler profiles. The CeT was chosen as the primary outcome due to its anatomical and technical advantages: it supplies the majority of foregut and is consistently well-visualized via the subcoastal window, allowing Doppler insonation with minimal angle correction.

#### Statistical analysis

Study data were collected and managed using REDCap electronic data capture tools hosted at the University of Oklahoma (NIH Award Number R25MD011564 and CTSA grant number UM1TR004403) [30, 31]. REDCap (Research Electronic Data Capture) is a secure, web-based software platform designed to support data capture for research studies, providing (1) an intuitive interface for validated data capture; (2) audit trails for tracking data manipulation and export procedures; (3) automated export procedures for seamless data downloads to common statistical packages; and (4) procedures for data integration and interoperability with external sources. Data were collected from the electronic medical record and analyzed using SPSS version 28 statistical software [IBM, Armonk, New York, USA]. Descriptive statistics were used for neonatal demographics and clinical data. The Shapiro-Wilk test was used to test continuous variables for normality. Mean (standard deviation) and median (interquartile range) were calculated for data with normal and non-normal distribution, respectively. Continuous variables were compared between the three groups according to PDA shunt pattern using parametric (one-way ANOVA) and non-parametric tests (Kruskal-Wallis) as appropriate. Binary group comparisons (groups 1 versus 2, 1 versus 3 and 2 versus 3) were done with parametric (Student *t* test) and non-parametric tests (Mann–Whitney) as appropriate when the three group analysis was significant (*p* > 0.05). Categorical variables were presented as frequencies (%) and compared using χ2 or Fisher’s exact test. Results were considered significant if *p* < 0.05. A convenience sample size was used and no power calculations were performed.

## Results

A total of 100 eligible patients were identified from the TNE databases at both centers, with 14 patients excluded for borderline PDA scores (between 6 and 7), presence of PFO/ASD > 3 mm and incomplete systemic Doppler data. A total of 86 patients were included in the final analysis with a median [IQR] gestational age and birth weight of 26.1 weeks [23.8–27.7] weeks and 767 [596, 1016] grams, respectively.

Table 1 depicts the demographic characteristics of the three groups. There was a higher rate of small for gestational age (SGA: birth weight less <3rd percentile for GA) infants in the No PDA group (*n* = 35) compared to the hsPDA (*n* = 34) and the Bidirectional PDA groups (n = 17) (p < 0.001). Table 2 depicts the clinical characteristics of the three groups. There were no differences in weight at TNE between the group; however, postmenstrual age was lower in the bidirectional PDA group compared to the hsPDA group (*p* < 0.05). There were no differences in respiratory severity score or lactic acid measured between the groups at the time of the TNE; however, the base deficit was notably greater in the bidirectional group (*p* < 0.001).

**Table 1:** Patient Demographics and clinical characteristics.

	No PDA (*n* = 35)	hsPDA left-to-right (*n* = 34)	PDA bidirectional (*n* = 17)	*p*
Male (percentage)	22 (62.9)	15 (44.1)	7 (41.2)	0.195
Birth weight (grams)	783 [560, 998]	765 [642, 1005]	668 [470, 1095]	0.764
Gestational age (weeks)	26.1 ± 2.5	26.1 ± 2.1	25.6 ± 3	0.841
Intrauterine growth restriction	8 (22.9)	2 (5.9)	4 (23.5)	0.107
Small for gestational age	13 (37.1) #	4 (11.8) *	6 (35.3)	0.040
Large for gestational age	0 (0)	1 (2.9)	3 (17.6)	0.015
Antenatal steroids				0.265
-None	3 (8.6)	7 (20.6)	2 (11.8)	
-Incomplete	9 (25.7)	13 (38.2)	4 (23.5)	
-Complete	23 (65.7)	14 (41.2)	11 (64.7)	
Cesarean section	23 (65.7)	23 (67.6)	12 (70.6)	0.939
Apgar at 1 minute	4 [3,5]	4 [1,6]	4 [2,5]	0.420
Apgar at 5 minutes	7 [7,8]	7 [4,8]	6 [5,7]	0.036

Neonatal demographics in the different groups. Results presented as frequencies (%), mean +/- SD and median [IQR]. # indicates a statistically significant difference (p < 0.05) between the No PDA and hsPDA groups. * indicates a statistically significant difference (p < 0.05) between the hsPDA and Bidirectional PDA groups.

**Table 2:** Clinical characteristics at time of TNE evaluation.

	No PDA (n = 35)	hsPDA left-to-right (n = 34)	PDA bidirectional (n = 17)	p
Age at the time of assessment (days)	7 [3,12]	7 [3,26] *	1 [0, 2]	<0.001
Postmenstrual age (weeks)	27.2 ± 2.5	27.7 ± 2.1 *	25.8 ± 3	0.071
Current weight (grams)	830 [560, 1085]	971 [777, 1147]	673 [455, 1090]	0.086
Ventilation mode				0.017
CPAP	6 (17.1) #	0 (0)	0 (0)	
Non-invasive NAVA	6 (17.1)	6 (17.6)	0 (0)	
Conventional MV	22 (5.7)	6 (17.6)	4 (23.5)	
HFJV or HFOV	21 (60)	22 (64.7)	13 (76.5)	
Mean airway pressure	8 [7,11] #	11 [9,13]	9 [8,11]	0.020
Fraction of inspired oxygen	25 [21,45]	31 [24,46]	38 [27,95]	0.017
Respiratory severity score	2.32 [1.47, 6.3]	3.56 [2.53, 6.96]	3.75 [2.52, 9.4]	0.106
Right arm systolic BP (mmHg)	70 ± 9 #	62 ± 13 *	53 ± 10	<0.001
Right arm diastolic BP (mmHg)	39 ± 11 #	29 ± 7	28 ± 10	0.001
Right arm mean BP (mmHg)	49 ± 9 #	40 ± 9	35 ± 9	<0.001
Lower extremity systolic BP (mmHg)	60 ± 12	55 ± 10 *	47 ± 7	0.002
Lower extremity diastolic BP (mmHg)	33 [26,42] #	23 [19,30]	19 [16,27]	<0.001
Lower extremity mean BP (mmHg)	42 ± 11 #	35 ± 8	30 ± 7 &	<0.001
Umbilical artery catheter systolic BP (mmHg)	51 [45,57] #	42 [40,45]	42 [34,50]	0.008
Umbilical artery catheter diastolic BP (mmHg)	32 ± 5 #	25 ± 6	25 ± 6	0.002
Umbilical artery catheter mean BP (mmHg)	38 [33,41] #	30 [27,34]	30 [24,37]	0.010
Hemoglobin (g/dL)	13.6 ± 1.7 #	12.4 ± 2.6	13.2 ± 3.5	0.160
pH	7.37 ± 0.06 #	7.31 ± 0.08	7.26 ± 0.11	<0.001
pCO2 (torr)	48.9 ± 9.6	50.3 ± 11.9	47.8 ± 10.5	0.718
pO2 (torr)	44 [35,60]	41 [32,52]	42 [38,48]	0.532
Oxygenation index	4.6 [3, 13.8]	7.3 [5.3, 16.8]	8.8 [6.2, 19.8]	0.177
Base deficit/excess	2.5 ± 3.4 #	0 ± 4.8 *	-5 ± 6.6	<0.001
Lactic acid (mmol/L)	1.75 [1.07, 2.77]	2.1 [1.1, 4.95]	2.9 [1.8, 7.9]	0.072
iNO administration	0 (0)	4 (11.8)	3 (17.6)	0.056
Vasopressor/ Inotrope administration	0 (0)	2 (5.9)	3 (17.6)	0.039

Results are presented in mean ± SD, median [IQR], n (%). CPAP: continuous positive airway pressure; NAVA: neutrally adjusted ventilatory assist; HFJV: high frequency jet ventilation; HFOV: high frequency oscillatory ventilation; PDA: patent ductus arteriosus; Respiratory severity score was calculated as mean airway pressure x fraction of inspired oxygen. BP: blood pressure; pCO2: partial pressure or carbon dioxide; pO2: partial pressure of oxygen; iNO: inhaled nitric oxide; TNE: targeted neonatal echocardiography; * If p < 0.05 comparing L-R PDA and bidirectional PDA; # if p < 0.05 comparing no PDA and L-R PDA.

Expected differences in echocardiography markers of hsPDA vs No PDA groups (Supplementary Table 2) included higher LVO/RVO, increased pulmonary vein D wave velocity, mitral valve E wave velocity, shorter IVRT, higher LA:Ao ratio, LV VTI and LVO (*p* < 0.001). There was no difference in right ventricular output (RVO) between the No PDA and hsPDA groups. A similar RVO in both groups possibly reflects maintained systemic blood flow in the hsPDA group in this cohort, while the increased LVO suggests increased pulmonary blood flow and pre-ductal flow, with increased Qp:Qs.

The primary outcome, absent or reversed diastolic flow in the CeT was present in 15 (44.1%), 2 (5.7%) and 1 (5.9%) of the patients in the hsPDA, No PDA and Bidirectional PDA groups respectively. Diastolic flow reversal was demonstrated in the DAo (87.9%), celiac artery (6.1%), SMA (6.7%) and MCA (2.9%) in the hsPDA group, but not in the No PDA or Bidirectional groups (Table 3). Infants in the hsPDA group had a higher incidence of absent diastolic blood flow in the MCA (*p* < 0.05) compared to the Bidirectional group.

**Table 3:** Systemic artery flow patterns and VTI in descending aorta, celiac artery (CeT), superior mesenteric artery (SMA) and middle cerebral artery (MCA).

Flow Pattern		No PDA	hsPDA	Bidirectional PDA
**Diastolic flow reversal, n (%)**	DAo	0	29 (87.9)	2 (11.8)
	CeT	0	2 (5.9)	0
	SMA	0	2 (5.9)	0
	MCA	0	1 (2.9)	0
**Absent diastolic flow, n (%)**	CeT	2 (5.9)	13 (38.2) ^#/*^	1 (5.9)
	SMA	7 (20)	18 (52.9)	9 (52.9)
	MCA	0	12 (35.3) ^#^	2 (11.8)
**Systemic Artery VTI (cm) [Min, Max]**	CeT	10.6 [8.9, 12.3]	7.8 [5.5, 9.6] #/*	12.8 [9.4, 20.2]
	SMA	7.9 [6, 10.2]	5.3 [3.7, 8.8] #	5.2 [3.2, 8.9] &
	MCA	5.5 [3.9, 6.9]	5.9 [4, 7.8]	4.4 [2.7, 6.8]

Results presented as frequencies (%) and median [IQR]. # indicates a statistically significant difference (p < 0.05) between the No PDA and hsPDA groups. * indicates a statistically significant difference (p < 0.05) between the hsPDA and Bidirectional PDA groups. & indicates a statistically significant difference (p < 0.05) between the no PDA and bidirectional group.

### Pre and post-ductal Dao

**Fig. 2:**
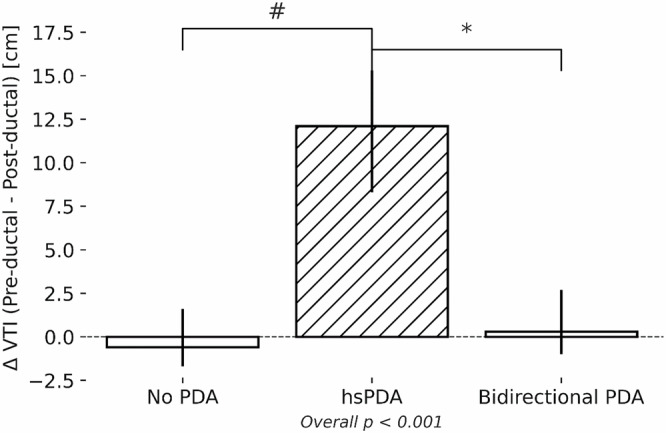
Difference between pre-ductal and post-ductal descending aortic velocity time integrals (Δ VTI) across PDA groups. **Caption:** # indicates a statistically significant difference (*p* < 0.05) between the no PDA and hsPDA groups. * indicates a statistically significant difference (*p* < 0.05) between the hsPDA and bidirectional PDA groups.

Doppler assessment of flow in the DAo showed that infants in the hsPDA group had a larger pre-ductal DAo VTI (*p* < 0.001); however, post-ductal DAo VTI was similar to the other two groups. Figure 2 illustrates the median [interquartile range] difference between pre-ductal descending aorta (DAo) VTI and net post-ductal DAo VTI (antegrade minus retrograde) in three groups: infants with no patent ductus arteriosus (PDA), infants with hemodynamically significant PDA (hsPDA), and those with a bidirectional PDA. A positive Δ VTI indicates higher antegrade flow proximal to the ductus, suggesting systemic steal through the PDA. Infants with hsPDA showed significantly higher Δ VTI values compared to both other groups. Retrograde flow in the post-ductal DAo was present in the hsPDA and bidirectional groups only. Infants with hsPDA exhibited a larger retrograde (diastolic) component, yielding a significantly lower net post‑ductal DAo VTI, consistent with greater diastolic steal (*p* < 0.001) and the difference between pre-ductal and post-ductal net DAo VTI (antegrade-retrograde) was higher in the hsPDA group (*p* < 0.001) only (Table 4).

**Table 4:** Echocardiographic markers for systemic Doppler comparisons.

	No PDA (n = 35)	hsPDA left-to-right (n = 34)	PDA bidirectional (n = 17)	p
Pre-ductal DAo VTI (cm)	8.6 ± 2.5 #	17.2 ± 4.5 *	9.6 ± 4.1	<0.001
Post-ductal DAo VTI (antegrade) (cm)	8.8 [6.5, 10.5]	8.7 [5.7, 10.3]	7.5 [4.5, 11.1]	0.443
Post-ductal DAo VTI (retrograde) (cm)	--	2.5 [2.2, 3.9]	0 [0, 0.1]	<0.001
Post-ductal DAo net VTI (antegrade – retrograde) (cm)	--	5.3 [3, 6.8]	7.6 [4.8, 11]	0.066
Difference between pre-ductal descending aorta VTI and post-ductal net descending aorta VTI (antegrade-retrograde) (cm)	-0.6 [-1.7, 1.6] #	12.1 [8.3, 15.3] *	0.3 [-1, 2.7]	<0.001
PFO/ASD	32 (91.4)	30 (90.9)	15 (88.2)	0.931
PFO/ASD size (mm)	1.5 [1.1, 1.9]	1.7 [1.2, 2.3]	1.9 [1.4, 2.1]	0.274
PFO/ASD direction				0.008
-Left-to-right	29 (90.6)	29 (96.7) *	9 (60)	
-Bidirectional	3 (9.4)	1 (3.3)	5 (33.3)	
-Right-to-left	0 (0)	0 (0)	1 (6.7)	
Reversal in the CeT (cm)	0 (0)	2 (6.1)	0 (0)	0.218
Absent diastolic flow in the CeT (cm)	2 (5.7) #	15 (44.1) *	1 (5.9)	<0.001
Celiac artery VTI (cm)	10.6 [8.9, 12.3] #	7.8 [5.5, 9.6] *	12.8 [9.4, 20.2]	<0.001
Celiac artery Vmax (m/s)	0.49 [0.42, 0.56]	0.5 [0.45, 0.64]	0.59 [0.51, 0.81]	0.085
Celiac artery Vmin (m/s)	0.14 [0.1, 0.17] #	0.06 [0, 0.08] *	0.15 [0.1, 0.28]	<0.001
Celiac artery Vmean (m/s)	0.29 [0.24, 0.32]	0.25 [0.21, 0.32] *	0.33 [0.26, 0.49]	0.015
Celiac artery PI	1.29 ± 0.41 #	1.83 ± 0.34 *	1.25 ± 0.43	<0.001
Celiac artery RI	0.69 [0.62, 0.8] #	0.89 [0.8, 1] *	0.67 [0.58, 0.8]	<0.001
Reversal in the SMA (cm)	0 (0) #	2 (6.7)	0 (0)	0.194
Absent diastolic flow in the SMA (cm)	7 (20)	20 (58.8)	9 (52.9) &	<0.001
Superior mesenteric artery VTI (cm)	7.9 [6, 10.2] #	5.3 [3.7, 8.8]	5.2 [3.2, 8.9] &	0.020
Superior mesenteric artery Vmax (m/s)	0.45 [0.34, 0.62]	0.48 [0.36, 0.67] *	0.37 [0.27, 0.47]	0.086
Superior mesenteric artery Vmin (m/s)	0.09 [0.21, 0.1] #	0 [0, 0.04]	0 [0, 0.09]	0.001
Superior mesenteric artery Vmean (m/s)	0.21 [0.16, 0.28]	0.23 [0.2, 0.3] *	0.18 [0.16, 0.24]	0.098
Superior mesenteric artery PI	1.74 ± 0.43	1.95 ± 0.39	1.78 ± 0.49	0.160
Superior mesenteric artery RI	0.8 [0.74, 0.95] #	1 [0.89, 1]	1 [0.77, 1]	<0.001
Reversal in the MCA (cm)	0 (0)	1 (2.9)	0 (0)	0.461
Absent diastolic flow in the MCA (cm)	0 (0) #	13 (38.2)	2 (11.8)	<0.001
Middle cerebral artery VTI (cm)	5.5 [3.9, 6.9]	5.9 [4, 7.8]	4.4 [2.7, 6.8]	0.194
Middle cerebral artery Vmax (m/s)	0.27 [0.21, 0.36] #	0.38 [0.31, 0.47] *	0.23 [0.14, 0.3]	<0.001
Middle cerebral artery Vmin (m/s)	0.06 [0.04, 0.08] #	0.04 [0, 0.06]	0.04 [0.02, 0.07]	0.015
Middle cerebral artery Vmean (m/s)	0.14 [0.1, 0.18] #	0.19 [0.15, 0.24] *	0.11 [0.07, 0.16]	<0.001
Middle cerebral artery pulsatility index	1.54 ± 0.38 #	1.78 ± 0.38	1.59 ± 0.46	0.045
Middle cerebral artery resistive index	0.79 [0.7, 0.82] #	0.88 [0.82, 1] *	0.78 [0.69, 0.85]	<0.001

Results presented as frequencies (%), mean +/- SD and median [IQR]. DAo: Descending aorta, VTI: velocity time integral, PFO: Patent foramen ovale, ASD: atrial septal defect, Vmax: maximum velocity, Vmin: minimum velocity, Vmean: mean velocity, CeT: celiac trunk, SMA: superior mesenteric artery, MCA: middle cerebral artery, PI: Pulsatility index, RI: resistive index. # indicates a statistically significant difference (p < 0.05) between the No PDA and hsPDA groups. * indicates a statistically significant difference (p < 0.05) between the hsPDA and Bidirectional PDA groups.

### Other indices of systemic blood

Infants in the hsPDA group had lower celiac artery VTI, compared to the other two groups (*p* < 0.05). SMA VTI was higher in the No PDA group compared to the other 2 groups (*p* < 0.05) Table 3. MCA VTI was similar in all groups. Celiac artery and SMA Vmax were comparable between groups; however, MCA Vmax was notably higher in the hsPDA group (*p* < 0.05). Vmin was consistently lower in the hsPDA group compared to the No PDA group in all systemic vessels (Table 4). MCA Vmean was higher in the hsPDA group (Table 4).

### Resistive and pulsatility Indices (RI and PI)

Table 4 depicts the CeT RI and PI were higher in the hsPDA compared to both other groups (*p* < 0.001). SMA RI was higher in the no PDA group while SMA PI was similar across all groups. Both MCA PI and RI were higher in the hsPDA group compared to the other groups (*p* < 0.05).

## Discussion

In this physiologic study of preterm infants, we demonstrated that hsPDA was associated with diastolic flow reversal in the post-ductal aorta, celiac artery trunk and superior mesenteric artery. However, contrary to our hypothesis, we found no significant differences in MCA diastolic flow or VTI across groups, irrespective of PDA magnitude. These flow patterns suggest that cerebral autoregulation mechanisms, possibly through adjustments in vascular resistance, are successful at preserving cerebral blood flow in this cohort. Previous studies have similarly found that superior vena cava (SVC) flow, a marker of upper body systemic blood flow, was also preserved and did not correlate with LVO or magnitude and direction of transductal shunts in preterm neonates [32, 33]. Interestingly, despite comparable post-ductal antegrade DAo VTI across all three groups, the net post-ductal DAo VTI was lower in the hsPDA group. Infants in the hsPDA group exhibited higher CeT and MCA RI and PI, which reflects the unique vascular regulatory mechanisms of the gut and brain [34]. VTI obtained via pulsed-wave Doppler represents the integral of blood velocity over the cardiac cycle (distance traveled per beat). When combined with vessel cross-sectional area and heart rate, VTI can be used to approximate stroke volume and flow. Although vessel diameters for the MCA, CeT, and SMA were not measured in our study, changes in VTI remain physiologically meaningful for evaluating relative flow differences. Conversely, PI and RI are indices of flow pulsatility and vascular resistance rather than absolute flow, which explains the differences observed between flow pattern classifications (Table 4) and VTI measurements. These complementary metrics should therefore be interpreted in the context of their distinct physiologic implications.

In the hsPDA group, absent or reversed diastolic blood flow in the SMA and CeT suggests compromised post-ductal systemic perfusion, often referred to as “ductal steal” [35]. Such hemodynamic alterations underscore the impact of hsPDA on systemic perfusion, particularly affecting the blood supply to vital abdominal organs Additionally, the net post-ductal descending aorta VTI (antegrade-retrograde) was lower in the hsPDA group, further supporting this theory. Notably, the difference in post-ductal descending aorta flow, including the presence of flow reversal and a better characterization of net distal flow through the calculation of pre- and post-ductal VTIs, is a novel finding that suggests lower systemic arterial blood flow. The observation that net post‑ductal DAo VTI was lower in the hsPDA group supports the concept that ductal steal predominantly compromises diastolic forward flow. By integrating both forward and reverse components, the net VTI provides a more physiologically meaningful representation of the effective forward perfusion pressure/velocity profile than antegrade VTI alone. Nonetheless, because VTI reflects velocity over time rather than absolute flow volume, and because we did not pair these measures with vessel diameter to derive flow, we interpret net DAo VTI as a relative hemodynamic marker rather than a quantitative measure of systemic blood flow. However, it is important to note that while Doppler measures velocity only, velocity does not equal volume – flow volume depends on both velocity and vessel cross sectional area, and confounding factors such as the unknown extent of regional micro-vascular responses could not be assessed.

Quantifying ductal shunt volume and systemic steal via echocardiography has known limitations. Normal, forward blood flow through vessels is driven by a pressure gradient – the difference between the upstream and downstream pressures. During systole, proximal pressure is higher than distal pressure, and it lowers during diastole. Absence or reversal of blood flow in diastole, indicates proximal/upstream pressure that drops lower than distal or downstream vascular bed. This implies that upstream pressure is insufficient to overcome the downstream resistance during the cardiac cycle in the downstream vascular bed [36]. While we cannot completely distinguish between flow-driven and resistance-driven bidirectional shunts, utilizing the PDA score alongside flow directionality provides valuable insights into the hemodynamic characteristics of these shunts. This combined approach enhances our understanding of the underlying mechanisms influencing shunt behavior.

Previous studies have described two mechanisms contributing to decreased blood flow in distal organs: in animal studies, reduced blood flow to the gastrointestinal blood vessels was driven by a local increase in vascular resistance. This correlates with our findings of increased PI and RI in the CeT, SMA and MCA. Human studies have reported increased PI and RI in the gut and cerebral arteries in infants with hsPDA [36].

### Splanchnic and cerebral circulation: connections to NEC, AKI and IVH

Multiple neonatal comorbidities including NEC, AKI and IVH have been linked to hsPDA with proposed mechanisms involving disruptions in blood flow in splanchnic, renal and cerebral circulation [37–39]. Our study provides further description of physiologic alteration to systemic circulatory patterns based on ductal shunt characteristics. Inadequate blood supply may facilitate bacterial translocation into the intestinal mucosa, contributing to necrotizing enterocolitis (NEC) [38, 40, 41]. However, evidence on altered splanchnic circulation patterns due to varying degrees of ductal shunt remains limited. Our findings indicate decreased perfusion in splanchnic circulation, as evidenced by elevated CeT pulsatility index (PI) and resistance index (RI), along with reduced post-ductal descending aorta (DAo) velocity-time integral (VTI) (*p* < 0.001), in the hsPDA group. Impaired perfusion may also lead to acute kidney injury (AKI) in premature infants with hsPDA, correlating with decreased renal flow. In our cohort, the net post-ductal DAo VTI was significantly lower (*p* < 0.001), suggesting decreased systemic perfusion, which could adversely affect renal blood flow in moderate to large patent ductus arteriosus (PDA). Near-infrared spectroscopy (NIRS) has also shown a relationship between reversed diastolic flow in the descending aorta and low mesenteric oxygenation, cerebral oxygenation and renal oxygenation [42–44]. In addition, preterm infants are at high risk for intraventricular hemorrhage (IVH) due to altered cerebral blood flow (CBF) linked to hsPDA [7, 45]. While indicators of increased cerebrovascular resistance, such as MCA RI and PI, were observed in the hsPDA group, MCA VTI remained similar. This distinction underscores the complexities of cerebral autoregulation, where increased resistance does not necessarily equate to reduced flow. These findings highlight the importance of interpreting Doppler parameters with a broader physiologic context and support the need for further research to explore confounding influences such as fetal inflammatory response and shunt volume heterogeneity. Importantly, they also point to the need for more advanced, physiology-driven to interrogate and potentially quantify cerebral perfusion in premature infants. Although Doppler ultrasound reflects blood flow velocity rather than absolute flow volume, characteristic patterns such as reversed end-diastolic flow are clinically meaningful and have been associated with adverse outcomes, including NEC.

Our study has several limitations. First, although we aimed to characterize the physiologic impact of the PDA shunt volume across the three groups, our analysis is limited to a single time point. Therefore, we are unable to assess the longitudinal relationship between physiological findings and clinical outcomes. These include post-ductal perfusion clinical parameters such as creatinine, levels, urinary output, NIRS, feeding tolerance, etc. Second, the timing of echocardiography assessments varied between the groups, with significant differences in postnatal age at the time of evaluation. The bidirectional group had lower median age at the time of assessment [1, (0,2)] days compared to infants in the hsPDA group 7, (3,26) days and No PDA group [7, (3,12) days] (*p* < 0.001). This variation limits comparability between the hsPDA group and the Bidirectional group. *Third*, we did not assess the impact of atrial level shunts on PDA score; while most patients had small atrial level shunts, this was not formally assessed.

## Conclusion

This retrospective physiologic study illustrates how varying degrees of ductal shunting influence systemic Doppler flow patterns in premature infants. The findings of diastolic flow reversal, reduced net post-ductal Dao VTI, decreased CeT VTI and elevated CeT RI/PI suggest a diminished capacity to maintain lower body blood flow in premature infants with hsPDA. However, the study was not designed to assess the clinical impacts of these flow alterations. Importantly, changes in systemic Doppler flow indices and single-artery measurements may not fully represent the overall end-organ perfusion. Further prospective studies are warranted to explore how these physiologic findings relate to clinical outcomes such as necrotizing enterocolitis, intraventricular hemorrhage and acute kidney injury.

## Supplementary information

Supplemental Table Legends

Supplemental Table 1

Suuplemental Table 2

## Data Availability

The datasets presented in this article are not publicly available because they contain de-identified data retrospectively collected from medical records, in accordance with local IRB regulations. Requests regarding dataset access should be directed to the corresponding author, Adrianne R. Bischoff—email: adrianne-rahdebischoff@uiowa.edu
