# Supplementary material for: Cerebral and intestinal Doppler patterns according to patent ductus arteriosus shunt characteristics in preterm infants
Source: J Perinatol. 2025 Nov 24;46(3):349–57. doi: 10.1038/s41372-025-02505-9 (PMC13008758; doi:10.1038/s41372-025-02505-9)
Supplement: Supplementary file 1 — Supplemental Table Legends [file 41372_2025_2505_MOESM1_ESM.docx]

**Supplemental Table 1**: Patent ductus arteriosus score, adapted from Rios *et al*.[25]

Caption: Supplemental Table 1: IVRT: isovolumic relaxation time; PV: pulmonary vein; LVO: left ventricular output; PDA: patent ductus arteriosus.

**Supplemental Table 2:** Echocardiographic markers for PDA assessment

**Caption:** Supplemental Table 2: Results are presented in mean± SD, median [IQR], n (%). PDA: patent ductus arteriosus; LV: left ventricle; VTI: velocity time integral; LVO: left ventricular output; RV: right ventricle; RVO: right ventricular output; R-L: right-to-left; L-R: left-to-right; * If p<0.05 comparing L-R PDA and bidirectional PDA; # if p<0.05 comparing no PDA and L-R PDA
