# Supplementary material for: Cerebral and intestinal Doppler patterns according to patent ductus arteriosus shunt characteristics in preterm infants
Source: J Perinatol. 2025 Nov 24;46(3):349–57. doi: 10.1038/s41372-025-02505-9 (PMC13008758; doi:10.1038/s41372-025-02505-9)
Supplement: Supplementary file 2 — Supplemental Table 1 [file 41372_2025_2505_MOESM2_ESM.docx]

**Supplemental Table 1**: Patent ductus arteriosus score, adapted from Rios *et al*.[25]

| **Marker** | **0 points** | **1 point** | **2 points** |
| --- | --- | --- | --- |
| Mitral valve E-wave velocity, cm/sec | <45 | ≥45 and <80 | ≥80 |
| IVRT, m/sec | >45 | >30 and ≤45 | <45 |
| PV D-wave velocity, cm/sec | <30 | ≥30 and <50 | ≥50 |
| Left atrium/aorta ratio | <1.3 | ≥1.3 and <2.2 | ≥2.2 |
| LVO, mL/min/kg | <250 | ≥250 and <430 | ≥430 |
| Diastolic flow reversal in descending aorta and/or celiac/middle cerebral artery | no | ----- | yes |
| PDA diameter[mm]/weight[kg] | <1.5 | ≥1.5 and <3 | ≥3 |
