# Supplementary material for: Cerebral and intestinal Doppler patterns according to patent ductus arteriosus shunt characteristics in preterm infants
Source: J Perinatol. 2025 Nov 24;46(3):349–57. doi: 10.1038/s41372-025-02505-9 (PMC13008758; doi:10.1038/s41372-025-02505-9)
Supplement: Supplementary file 3 — Suuplemental Table 2 [file 41372_2025_2505_MOESM3_ESM.docx]

**Supplemental Table 2:** Echocardiographic markers for PDA assessment

|  | No PDA (n=35) | hsPDA  left-to-right (n=34) | PDA bidirectional (n=17) | p |
| --- | --- | --- | --- | --- |
| Pulmonary vein D wave (cm/s) | 29 [23, 33] # | 48 [38, 59] * | 27 [21, 30] | <0.001 |
| Mitral valve E wave (cm/s) | 41 [36, 47] # | 62 [56, 74] * | 37 [27, 47] | <0.001 |
| Isovolumic relaxation time (msec) | 47.4 ± 13.1 # | 26.7 ± 7.5 | 51.9 ± 13.7 | <0.001 |
| LV VTI (cm) | 8.6 [7, 9.1] # | 12.3 [11.1, 15.4] * | 8.1 [6.1, 9.9] | <0.001 |
| Heart rate (bpm) | 160 [149, 173] | 162 [153, 170] * | 148 [142, 160] | 0.031 |
| Left ventricular outflow tract diameter (cm) | 0.37 [0.32, 0.42] # | 0.41 [0.37, 0.45] * | 0.31 [0.28, 0.39] | <0.001 |
| Left ventricular output (mL/kg/min) | 167.4 ± 44 # | 302.5 ± 70.6 * | 137.8 ± 50.4 | <0.001 |
| Low LVO (<150 mL/min/kg) | 11 (31.4) | 1 (2.9) | 10 (58.8) | <0.001 |
| Very low LVO (<100 mL/min/kg) | 3 (8.6) | 0 (0) | 5 (29.4) | 0.003 |
| High LVO (>250 mL/min/kg) | 1 (2.9) | 23 (67.6) | 0 (0) | <0.001 |
| LA:Ao ratio | 1.33 ± 0.29 # | 1.87 ± 0.33 | 1.41 ± 0.39 | <0.001 |
| Right ventricular outflow tract diameter (cm) | 0.38 ± 0.05 # | 0.43 ± 0.06 * | 0.36 ± 0.07 | <0.001 |
| RV VTI (cm) | 8.53 ± 1.95 # | 6.96 ± 1.93 * | 5.57 ± 1.9 | <0.001 |
| Right ventricular output (mL/min/kg) | 196.4 ± 56.2 | 174.2 ± 53.5 * | 114.1 ± 48.3 | <0.001 |
| LVO/RVO ratio | 0.87 ± 0.17 # | 1.87 ± 0.57 * | 1.26 ± 0.34 | <0.001 |
| Holodiastolic flow reversal in the post-ductal descending aorta | 0 (0) # | 29 (87.9) * | 2 (11.8) | <0.001 |
| PDA size (mm) | -- | 2.1 [2, 2.6] | 1.9 [1.6, 3] | 0.488 |
| PDA size indexed to infant’s weight (mm/kg) | -- | 2.4 [2, 2.8] | 2.6 [1.9, 3.6] | 0.201 |
| Percentage of transductal VTI spent with R-L flow | -- | 9.8 ± 9.7 | 39.9 ± 22.9 | 0.020 |
| ≥30% of VTI spend R-L flow | -- | 0 (0) | 11 (64.7) | 0.035 |
| PDA score | -- | 9 [9, 10] | 3 [2, 4] | <0.001 |
